# Prognostic Value and Clinicopathological Differences of HIFs in Colorectal Cancer: Evidence from Meta-Analysis

**DOI:** 10.1371/journal.pone.0080337

**Published:** 2013-12-06

**Authors:** Zhigang Chen, Xin He, Wenjie Xia, Qi Huang, Zhigang Zhang, Jun Ye, Chao Ni, Pin Wu, Dang Wu, Jinghong Xu, Fuming Qiu, Jian Huang

**Affiliations:** 1 Department of Oncology, Second Affiliated Hospital, Zhejiang University School of Medicine, Hangzhou, China; 2 Cancer Institute (Key Laboratory of Cancer Prevention & Intervention, National Ministry of Education, Provincial Key Laboratory of Molecular Biology in Medical Sciences), Zhejiang University School of Medicine, Hangzhou, China; 3 Department of Hematology, Second Affiliated Hospital, Zhejiang University School of Medicine, Hangzhou, China; 4 Department of Pathology, Second Affiliated Hospital, Zhejiang University School of Medicine, Hangzhou, China; University General Hospital of Heraklion and Laboratory of Tumor Cell Biology, School of Medicine, University of Crete, Greece

## Abstract

**Background:**

The prognostic value of HIFs in colorectal cancer was evaluated in a large number of studies, but the conclusions were inconclusive. Meanwhile, clinicopathologic differences of HIF-1α and HIF-2α were rarely compared in recent studies.

**Methodology:**

Identical search strategies were used to search relevant literatures in the PubMed and Web of Science databases. The prognostic significances and clinicopathological differences of HIFs in CRC were analyzed.

**Principal Findings:**

A total of 23studies comprising 2984 CRC patients met the inclusion criteria. The results indicated that overexpressed HIFs were significantly associated with increase of mortality risk, including overall survival (OS) (HR 2.06 95%CI 1.55–2.74) and disease free survival (HR 2.84, 95%CI 1.87–4.31). Subgroup analysis revealed that both overexpressed HIF-1α and HIF-2α had correlations with worse prognosis. The pooled HRs were 2.01 (95% CI: 1.55–2.6) and 2.07(95% CI: 1.01–4.26). Further subgroup analysis on HIF-1α was performed by study location, number of patients, quality score and cut-off value. The results showed that HIF-1α overexpression was significantly associated with poor OS, particularly in Asian countries (HR 2.3, 95% CI: 1.74–3.01), while not in European or other countries. In addition, overexpression of HIF-1α was closely related with these clinicopathological features, including Dukes' stages (OR 0.39, 95% CI: 0.17–0.89), UICC stages (OR 0.42 95% CI: 0.3–0.59), depth of invasion (OR 0.71, 95% CI: 0.51–0.99), lymphnode status (OR 0.49, 95% CI: 0.32–0.73) and metastasis (OR 0.29, 95% CI: 0.11–0.81). While overexpression of HIF-2α was only associated with grade of differentiation (OR 0.48, 95% CI: 0.29–0.81).

**Conclusions:**

This study showed that both HIF-1α and HIF-2α overexpression were associated with an unfavorable prognosis. HIF-1α overexpression seemed to be associated with worse prognosis in Asian countries. Additionally, HIF-1α and HIF-2α indicated distinct clinicopathologic features.

## Introduction

Colorectal cancer is the third most common malignancy worldwide, and one of the leading causes of cancer-related deaths [1]. An increasing trend in the incidence of this carcinoma has been noticed in the Asian nations. Despite recent therapeutic advances, its 5-year survival rate is still pessimistic due to its recurrence and drug resistance [2]. Growing evidence suggests that hypoxia plays a pivotal role in disease progression and therapy resistance in most solid tumors, including colorectal cancer [3], [4]. Rapid oxygen consumption and aberrant tumor angiogenesis and blood flow result in a hypoxic tumor environment. Owing to the fundamental importance of oxygen for metabolism and survival, cells have evolved intricate response mechanisms to respond to hypoxia. The most important regulators mediating the primary transcriptional responses to hypoxic stress are hypoxia-inducible factors (HIFs). Given that hypoxia promotes tumor progression and therapy resistance, HIFs are expected to be useful biomarkers associated with progress disease and poor prognosis in CRC. Increased expression of HIFs has also been observed in a broad range of human cancer cell types, and has been associated with poor prognosis in many cases [5], but the prognostic value of HIFs for CRC patients is inconclusive.

HIFs are heterodimers composed of an inducible a-subunit (HIF-1α, HIF-2α or HIF-3α), and a constitutive HIF-1β subunit (also known as aryl hydrocarbon receptor nuclear translocator or ARNT), which together form the HIF-1, HIF-2 and HIF-3 transcriptional complexes, respectively. Of the three HIF family members, HIF-1 and HIF-2 are the most well-characterized. HIF-1α and HIF-2α are usually detected to measure tumor oxygen levels because the HIF-1β subunit is constitutive. HIF-1α and HIF-2α have 48% amino acid sequence identity and similar protein structures, but distinct target genes and mechanisms of regulation. HIF-1α preferentially induces glycolytic pathway, whereas HIF-2α regulates genes involved in tumor growth, cell cycle and maintaining stem cell pluripotency [6]. Thus, HIF1α and HIF2α can promote highly divergent, even opposing, outcomes, which results in distinct clinicopathologic features and prognosis. Multiple xenograft tumour models also support the hypothesis that HIF1α and HIF2α play different roles in tumor progression by regulating both shared and unique target genes [7]. However, clinicopathologic and prognostic differences of HIF1α and HIF2α in CRC were rarely compared in recent studies.

Therefore, we made a meta-analysis from eligible studies to investigate the relationship between HIF expression and prognosis of CRC patients. Meanwhile, we performed a subgroup analysis to assess the roles of HIF-1α and HIF-2α in clinicopathologic features and prognosis of CRC.

## Materials and Methods

### Identification and eligibility of relevant studies

We searched literature from PubMed, WanFang and Web of Science databases using the terms: “HIF”, “colorectal neoplasms”, “colorectal Cancer”, “colon cancer” “rectal cancer”, “prognosis” with all possible combinations. Bibliographies, review articles and other pertinent studies were searched manually for additional eligible studies.

The inclusion criteria for eligibility of a study in the meta-analysis were as follows: (1) evaluating HIF expression in the human CRC tissues; (2) assessing the relationships between HIFs expression with CRC clinicopathologic features or prognosis; (3) articles written in English or Chinese; (4) sufficient information provided to estimate hazard ratio (HR) about overall survival (OS) or disease free survival (DFS), or to estimate odds ratio (OR) about clinicopathologic features. In addition, letters, reviews, conference abstracts, and case reports were not in the scope of our analysis because of the limited data. Overlapping articles were also excluded from this meta-analysis, only the most recent or the most complete study was involved in the analysis.

### Data extraction and management

Two investigators (Xin He and Wenjie Xia) reviewed each eligible study independently and extracted data from all the publications meeting the inclusion criteria. Controversial problems were arbitrated by the third investigator (Jinhong Xu). The following information was collected from each study: the first author's name, year of publication, country of origin, number of patients, gender of patients, HIF isoforms, source and dilution of antibody, cut-off value, tumor characteristics, condition of adjuvant therapy and survival data.

### Methodological assessment

Newcastle–Ottawa quality assessment scale (NOS) was used to assess the quality of each study [8]. The score assessed eight items of methodology, categorized into three dimensions including selection, comparability, and outcome. A maximum of 1 score was awarded for each item with the exception of the item related to comparability that allowed the assignment of two scores. A total of 0 and 9 scores were respectively designated as lowest and highest quality, and the studies with 6 scores or more were graded as the high quality ones in the scale. The scores provided by two researchers were compared and a consensus value for each item was achieved.

### Statistical methods

For the pooled analysis of the impact of HIF expression on survival outcome, HRs and its 95% CI were used. If these statistical variables were described in a literature, we pooled it directly; otherwise, they were calculated from available numerical data in the articles according to the methods described by Parmar [9]. In brief, if the trials offered the data such as log-rank test p values, number of total events. The number of aberrant HIF expression and number of preserved HIF expression were extracted to allow estimation of the HR and its 95% CI. If only Kaplan Meier graphs were published, Kaplan-Meier curves were read by Engauge Digitizer version4.1 (http://digitizer.sourceforge.net/). Time-to-event data from the Kaplan–Meier curves was extracted and HR and its 95% CI were calculated via SPSS16.0. Odds ratios (ORs) and their 95%CIs were combined to evaluate the association between HIF expression and clinicopathological factors, such as differentiation grade, Dukes' stages, depth of invasion, lymphnode status and metastasis. An observed HR>1 implies worse survival for the group with overexpressed/negative HIF expression. An observed OR<1 implies unfavorable parameters for the group with overexpressed/negative HIF expression. The impact of overexpressed/negative HIF expression on survival or clinicopathological factors was considered to be statistically significant if the 95%CI did not overlap with 1. Heterogeneity in between-study was assessed by Chi- square based Q statistical test [10]. And the I^2^ statistic to quantify the proportion of the total variation, which is due to inter-study heterogeneity rather than sampling error and is measured from 0% to 100% [11]. Higher values indicate a greater degree of heterogeneity. When the studies were found to be homogeneous(with *P*>0.10 for the Q test), the pooled ORs and HRs estimate of each study were calculated by the fixed-effects model (the Mantel-Haenszel method) [12]. Otherwise, we chose the random-effects model (the DerSimonian and Laird method) [13]. We assessed the possibility of publication bias by visually assessing a funnel plot for asymmetry and by quantitatively performing Egger's test. Publication bias was indicated when p value of Egger's test <0.05. The meta-analysis was performed using STATA version 12.0 software (Stata Corporation, Collage Station, Texas, USA). All the P values were for a two-side test and considered statistically significant when p<0.05.

## Results

### Description of studies

**Figure 1:**
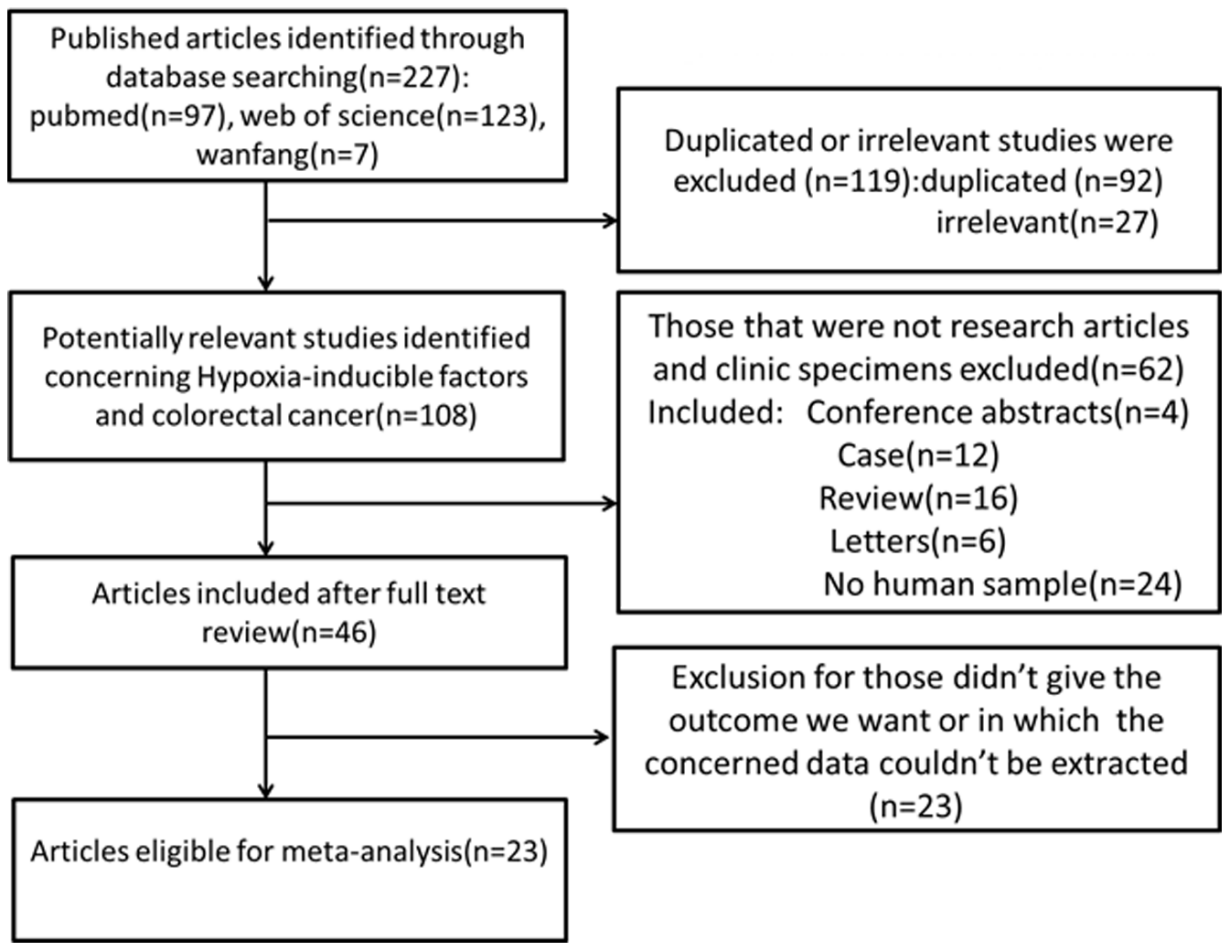
Flow diagram of study selection procedure.

As shown in **Figure 1**, 227 published records were identified from a search of the above databases using the search strategy as described above. After exclusion of the studies that were out of the scope of our systematic review, a total of 23 eligible studies were included in the final meta-analysis [4], [5], [14]–[32]. Of these 23 publications, 20 studies assessed the relationships between HIF-1 expression with CRC clinicopathologic features or prognosis, while 6 studies evaluated the association of HIF-2 expression and CRC pathological features or prognosis. The clinical features of these 23 included studies were summarized in **Table 1**. These studies were published from 2003 to 2013, and total 2984 CRC patients were enrolled. Sample sizes ranged from 30 to 731 patients (mean 130). 14 of these studies enrolled less than 100 patients and 9 studies included more than 100 patients. 6 of these studies evaluated patients from China, 5 from Japan, 3 from England, others from America, Korea, Finland, Germany, Austrialia, Holand and Greece. 19 of these studies got 6 scores or more in methodological assessment, which meant they had high qualities.

**Table 1:** Characteristics of studies included in the meta-analysis.

First author	Year	Country	HIF isoforms	Number of total patient(positive)	Expression location	Method	Antibody source	Antibody dilution	Definition of HIF positive	HR estimation	Quality score
Xie	2012	China	HIF-1α	93(46)	C and N	IHC	Santa Cruz	1∶100	Multiplying the intensitys core by expressions core≥median value	NA	8
Korkeila	2011	Finland	HIF-1α	168(68)	N	IHC	BD	1∶100	Weak,moderate or strong staining	HR for DFS	7
Shioya	2011	Japan	HIF-1α	50(21)	N	IHC	Neomarkers	1∶20000	40%	HR for OS and survival curves for DFS	8
Havelund	2011	Denmark	HIF-1α	86(39)	N	IHC	BD	1∶75	Summing the intensitys core and expressions core≥3	Survival curves for OS	7
Saigusa	2011	Japan	HIF-1α	52(NA)	NA	RT-PCR	NA	NA	0.0212 for PFS and 0.1274 for OS	HR for OS and DFS	7
Mohammed	2011	Spain	HIF-2α	154(NA)	NA	RT-PCR	NA	NA	NA	NA	6
Kwon	2010	Korea	HIF-1α	311(196)	N	IHC	Novus	1∶50	10%	HR for OS and DFS	7
Wu	2010	China	HIF-1α	68(30)	C and N	IHC	Abcam	1∶200	Multiplying the intensitys core by expressions core≥5	NA	6
Zheng	2010	China	HIF-1α	62(39)	C and N	IHC	Boster	1∶400	Summing the intensitys core and expressions core≥5	NA	5
Baba	2010	United States	HIF-1α	731(142)	C	IHC	Santa Cruz	1∶500	Moderate staining>50% or Any strong staining	HR for OS	7
			HIF-2α	695(322)	C	IHC	Santa Cruz	1∶250	Weak to strong expression.	HR for OS	
Toiyama	2010	England	HIF-1α	40(NA)	NA	RT-PCR	NA	NA	0.3649	HR for DFS	5
Gao	2009	China	HIF-1α	71(39)	C and N	IHC	Zymed	1∶50	Any staining	HR for OS	8
Jubb	2009	Austrialia	HIF-2α	155(60)	N	IHC	BD	NA	Any staining	HR for OS	6
Rajaganeshan	2009	England	HIF-1α	55(25)	C or N	IHC	Abcam	1∶100	>10% in nuclear or distinct, Strong staining in cytoplasm	HR for DFS	4
Schmitz	2009	Germany	HIF-1α	129(34)	N	IHC	Transduction Laboratories	1∶10	Any staining	NA	8
Rasheed	2009	England	HIF-1α	90(48)	C and N	IHC	Novus	1∶500	NA	HR for DFS	7
			HIF-2α	90(58)	C and N	IHC	Novus	1∶100	NA	NA	
Cleven	2007	Holand	HIF-2α	133(110)	N	IHC	BD	1∶120	5%	HR for OS	4
Lu	2006	China	HIF-1α	30(19)	N	IHC	NA	1∶200	10%	Survival curves for OS	7
Theodoropoulos	2006	Greece	HIF-1α	92(44)	N	IHC	Stress Gene	1∶1200	Moderate or strong staining	HR for OS and DFS	7
Yoshimura	2004	Japan	HIF-1α	87(39)	N	IHC	Novus	1∶1000	5%	Survival curves for OS	6
			HIF-2α	87(26)	N	IHC	Novus	1∶1000	5%	Survival curves for OS	
Kuwai	2003	Japan	HIF-1α	139(81)	C and N	IHC	Dako	1∶1000	9.60%	NA	6
Shimomura	2013	Japan	HIF-1α	64(20)	C	IHC	Novus	1∶50	Sum the intensity and percentage scores≥6	Survival curves for OS	7
Yu	2012	China	HIF-1α	124(67)	C	IHC	Abcam	1∶100	10%	Survival curves for OS	6

**NA, not available; HR, hazard ratio; OS, overall survival; DFS, disease free survival**; **IHC**, **immunohistochemistry**; C, cytoplasm; N, nucleus.

### Impact of HIFs expression on overall survival and disease free survival of colorectal cancer

**Figure 2:**
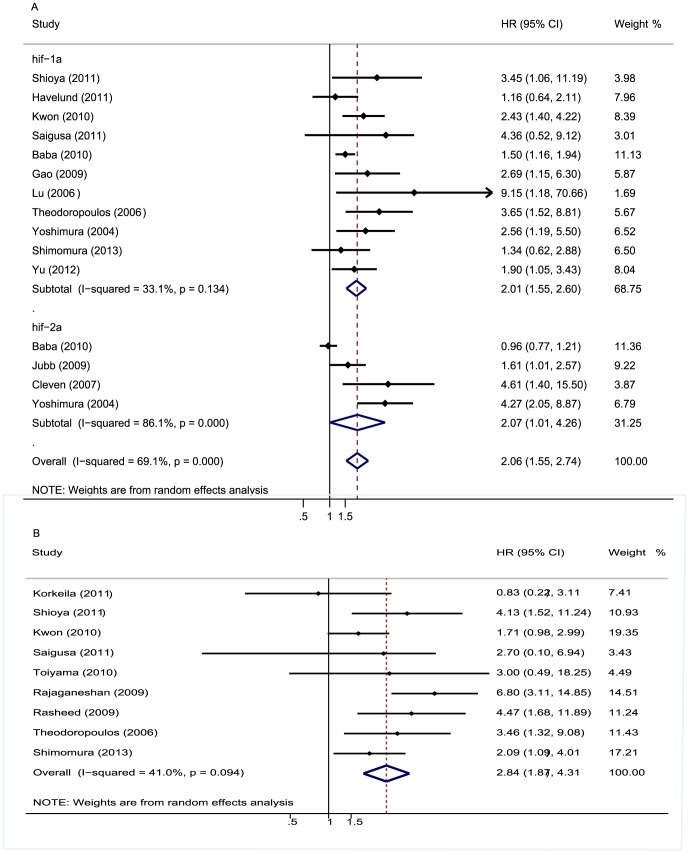
Forrest plot of Hazard ratio (HR) for the association of different HIF isoforms expression with overall survival (OS) and disease free survival (DFS). **A.** HRs with corresponding 95% CIs of the HIFs expression with OS. **B.** HRs with corresponding 95% CIs of the HIFs expression with DFS. HR>1 implied worse survival for the group with increased HIFs/negative expression and overexpressed HIFs was significantly with the worse prognosis of CRC patients.

**Figure 3:**
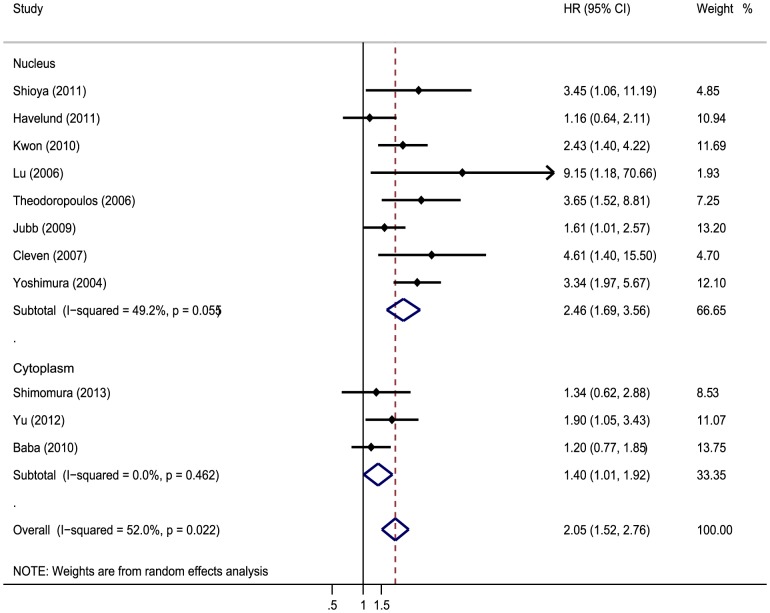
Forrest plot of Hazard ratio (HR) for the association of HIF in different subcellular localization with overall survival (OS).

The meta-analysis was performed on 15 studies assessing the association of HIFs expression with OS. The pooled HR was 2.06 (95%CI 1.55–2.74; I^2^ 69.1%) (**Figure 2A**). Nine studies evaluating the correlation of HIFs expression with DFS were all about HIF-1α. The pooled HR was 2.84 (95%CI 1.87–4.31; I^2^ 41%) (**Figure 2B**). It suggested that overexpressed HIF was significantly associated with increase of mortality risk. In addition, sensitive analysis was performed. We removed one study at a time and evaluated the rest, pooled HR of HIFs overexpression on OS ranged from 1.98(95% CI: 1.5–2.61) to 2.28(95% CI: 1.74–2.98) (**Table 2**), and combined HR of HIFs overexpression on DFS ranged from 2.34(95% CI: 1.68–3.26)to 3.22(95% CI: 2.08–4.99) (**Table 3**). We also performed subgroup analysis about association of HIFs expression with OS by HIF isoforms, the results showed that both HIF-1α and HIF-2α were associated with worse prognosis. The pooled HR was 2.01 (95% CI: 1.55–2.6, I^2^ 33.1%) and 2.07(95% CI: 1.01–4.26, I^2^ 86.1%) respectively (**Figure 2A**). Subgroup analysis about association of different subcellular localization of HIFs expression with OS was performed, and the results showed that the correlation was not changed no matter where HIF located in (nucleus or cytoplasm). The pooled HR was 2.456 (95% CI: 1.694–3.561, I^2^ 49.2%) and 2.049(95% CI: 1.519–2.764, I^2^ 0%), respectively (**Figure 3**).

**Table 2:** HRs (95% CI) of sensitivity analysis for HIFs overexpression on OS.

Study omitted	Estimated HR	low value of 95%CI	High value of 95%CI
Korkeila (2011)	3.08576	2.080869	4.575933
Shioya (2011)	2.716886	1.716758	4.299657
Kwon (2010)	3.219644	2.075922	4.993494
Saigusa (2011)	2.852396	1.829702	4.446713
Toiyama (2010)	2.839389	1.815004	4.441936
Rajaganeshan (2009)	2.338718	1.678648	3.258336
Rasheed (2009)	2.684669	1.706306	4.224007
Theodoropoulos (2006)	2.776092	1.734063	4.444295
Shimomura (2013)	3.028906	1.852393	4.95266
Combined	2.8408349	1.8734429	4.3077604

**Table 3:** HRs (95% CI) of sensitivity analysis for HIFs overexpression on DFS.

Study omitted	Estimated HR	low value of 95%CI	High value of 95%CI
Shioya (2011)	2.0867274	1.567879	2.7772751
Havelund (2011)	2.2600963	1.7024611	3.0003829
Kwon (2010)	2.1164002	1.5588678	2.8733358
Saigusa (2011)	2.0795095	1.5696353	2.7550092
Baba (2010)	2.2764547	1.7367126	2.9839401
Gao (2009)	2.1033344	1.5671818	2.8229115
Lu (2006)	2.0664124	1.5748442	2.7114177
Theodoropoulos (2006)	2.0455844	1.541568	2.7143891
Yoshimura (2004)	1.9817994	1.5027167	2.6136189
Shimomura (2013)	2.2203045	1.6552455	2.97826
Yu (2012)	2.1814032	1.602671	2.9691179
Jubb (2009)	2.2353566	1.6377747	3.0509808
Cleven (2007)	2.0521758	1.554372	2.7094064
Combined	2.127789	1.6131618	2.8065913

Moreover, further subgroup analysis on HIF-1α was performed by study location, number of patients, antibody dilution, cut-off value. Subgroup analysis indicated a significant relation between HIF-1α overexpression and OS was exhibited in Asian countries (HR 2.3, 95% CI: 1.74–3.01, I^2^ 0%). Other factors comprising number of patients, antibody dilution and cut-off value did not alter the significant OS of overexpressed HIF-1α (**Table 4**).

**Table 4:** Stratified analysis of pooled hazard ratios for colorectal cancer patients with overexpressed HIF-1α.

					Heterogeneity		
Stratified analysis	Number of studies	Number of patients	Pooled HR(95%CI)	P value	I^2^(%)	P value	Model used
Study location							
Asia	8	789	2.3(1.74–3.01)	0.000	0	0.598	FEM
Europe	2	178	1.96(0.64–6.03)	0.239	77.7	0.034	REM
Nubmer of patients							
>100	3	1166	1.67(1.34–2.07)	0.000	23.3	0.272	FEM
<100	8	532	2.11(1.54–2.88)	0.000	35.7	0.144	FEM
Cut off value							
Percentage	5	602	2.41(1.72–3.38)	0.000	0	0.621	FEM
Staining	2	163	3.12(1.69–5.75)	0.000	0	0.624	FEM
Percentage+staining	3	881	1.43(1.14–1.79)	0.002	0	0.727	FEM
Dilution							
≤1∶500	6	686	1.85(1.39–2.46)	0.000	29.1	0.217	REM
>1∶500	4	960	1.73(1.37–2.17)	0.000	52.2	0.099	FEM

REM, random-effectsmodel; FEM, fixed-effectsmodel; HR, hazard ratio; CI, confidenceinterval.

### Correlation of HIFs expression with clinicopathological parameters

**Figure 4:**
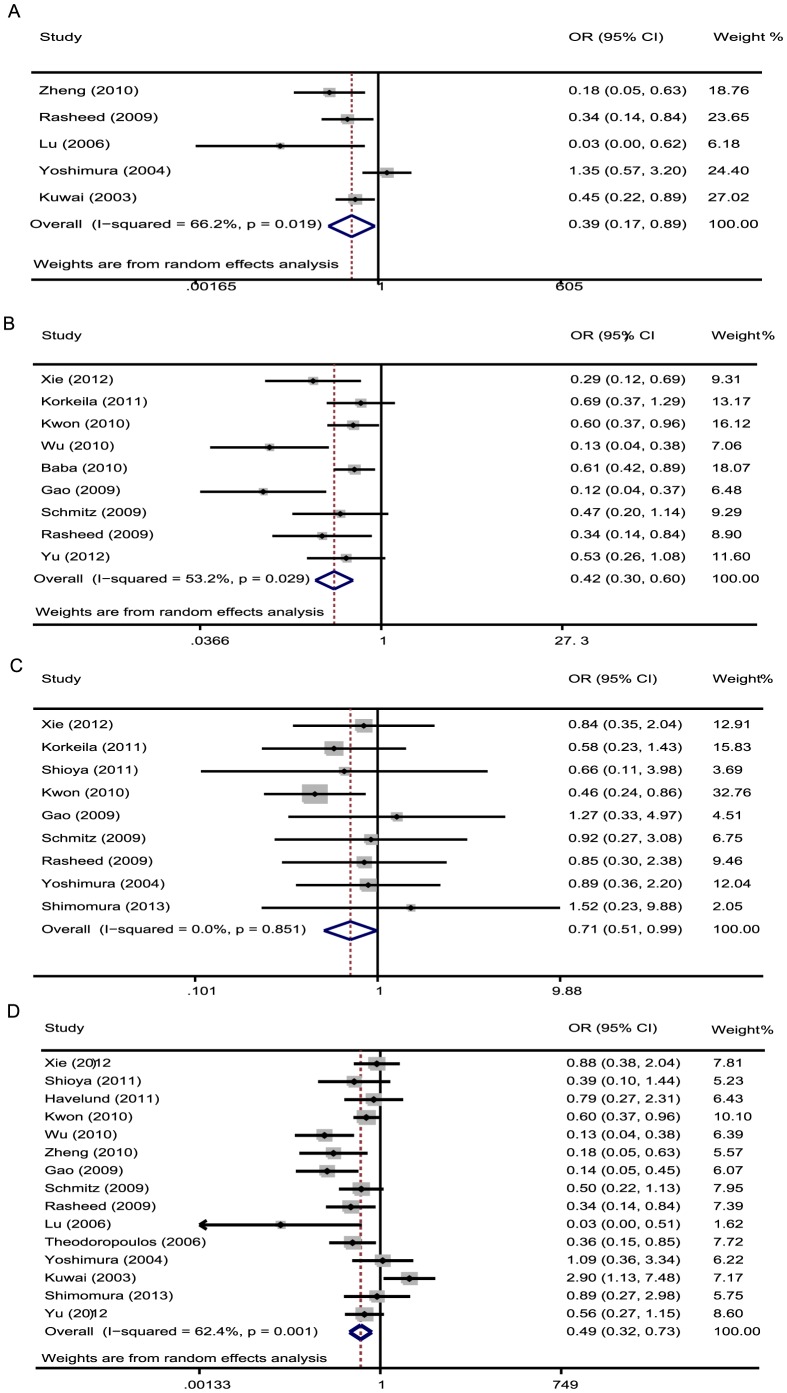
Forrest plot of odds ratios (ORs) for the association of HIF-1α expression with clinicopathological features. **A.** ORs with corresponding 95% CIs of the HIF-1α expression with Dukes' stages. OR<1 suggested that unfavorable parameters for the group with increased HIF-1α expression/negative. Overexpressed HIF-1α was associated with advanced Dukes' stage of CRC. **B.** ORs with corresponding 95% CIs of the HIF-1α overexpression with UICC stage. OR<1 suggested that unfavorable parameters for the group with increased HIF-1α expression/negative and HIF-1α overexpression was associated with advanced stage of CRC. **C.** ORs with corresponding 95% CIs of the HIF-1α overexpression with depth of invasion. **D.** ORs with corresponding 95% CIs of the HIF-1α overexpression with lymphnode metastasis.

The meta-analysis was also assessed the correlation between HIF-1α expression and clinicopathological characteristics of CRC. As shown in **Table 5**, overexpression of HIF-1α was significantly associated with Dukes' stages (OR 0.39, 95% CI: 0.17–0.89), UICC stages (OR 0.42 95% CI: 0.3–0.59), depth of invasion (OR 0.71, 95% CI: 0.51–0.99), lymphnode status (OR 0.49, 95% CI: 0.32–0.73) and metastasis (OR 0.29, 95% CI: 0.11–0.81). Furthermore, there was no significant association between HIF-1α expression with grade of differentiation. The pooled OR was 0.97 (95% CI: 0.67–1.39). (**Figure 4**).

**Table 5:** HIF-1α and HIF-2α expression and clinicopathological features for colorectal cancer.

					Heterogeneity		
Clinicopathological features	Nuber of studies	Nuber of patients	Pooled OR (95%CI)	P value	I^2^(%)	P value	Model used
HIF-1α							
Differentiation grade	15	2226	0.97 (0.67–1.39)	0.864	39.2	0.06	REM
Dukes' stages	5	408	0.39(0.17–0.89)	0.025	66.2	0.019	REM
Lymphnode status	15	1490	0.49(0.32–0.73)	0.001	62.4	0.001	REM
Metastasis	5	480	0.29(0.11–0.81)	0.018	52.8	0.076	REM
UICC stage	9	1733	0.42(0.3–0.59)	0.000	53.2	0.029	REM
Depth of invasion	9	1016	0.71(0.51–0.99)	0.045	0.00	0.851	FEM
HIF-2α							
Differentiation grade	2	782	0.484(0.289–0.812)	0.006	58	0.123	FEM
Dukes' stages	2	177	0.9(0.197–4.168)	0.9	82	0.019	REM
Lymphnode status	3	329	0.95(0.418–2.16)	0.904	63.7	0.064	REM
Depth of invasion	2	177	0.379(0.038–3.798)	0.409	83.4	0.014	REM

REM, random-effects model; FEM, fixed-effects model; OR, odds ratio; CI, confidence interval.

In addition, we evaluated the correlation between HIF-2α overexpression with clinicopathological characteristics of CRC. The result showed that overexpression of HIF-2α was significantly associated with grade of differentiation (OR 0.48, 95% CI: 0.29–0.81). There was no significant association between HIF-2α expression with Dukes' stages, depth of invasion and lymphnode status. The pooled OR was 0.91(95% CI: 0.20–4.17), 0.38 (95% CI: 0.04–3.80), and 0.95 (95% CI: 0.428–2.16), respectively (**Table 5**).

### Publication bias

Egger's test indicated that there was no evidence of significant publication bias after assessing the funnel plot (**Figure S1–S3**) for the studies included in our meta-analysis.

## Discussion

Hypoxia has been recognized as a common feature of solid tumors and a negative prognostic factor for response to treatment and survival of cancer patients. In 1993, Höckel reported that cervix cancer patients with hypoxic tumors (median pO_2_<10 mmHg) had a significantly lower overall and recurrence-free survival [33]. Since then, hypoxia has been found to indicate a highly aggressive disease phenotype associated with poor prognosis in many cancers, including brain, breast, prostate, pancreas, cervix, bladder and ovary [34]–[37]. HIFs are the best characterized markers mediating transcriptional responses to hypoxic stress and expected to be unfavorable prognostic indicators. Hypoxia and consequently HIF activation is regarded as an important stimulus of CRC angiogenesis. HIF binds to the HRE in the VEGF promoter region, leading to up- regulation of VEGF transcription and the formation of new blood vessels [38]. Surprisingly, both HIF-1 and HIF-2 can function as tumor suppressors in certain cancers [39]. Many studies were also performed to assess the prognostic value of HIF for CRC patients, but the conclusions were also inconclusive. On the other hand, HIF-1 and HIF-2 have distinct target genes, but few studies compared the clinicopathologic and prognostic differences between HIF-1 and HIF-2.

This meta-analysis aimed to examine the association between HIFs expression and the prognosis of CRC patients, and assess the roles of HIF-1α and HIF-2α in clinicopathologic features. Our analysis combined the outcomes of 23 studies comprising 2984 CRC patients, indicating that overexpressed HIF was significantly associated with increase of mortality risk, including OS (2.06 95%CI 1.55–2.74; Z = 4.95; P = 0.000) and DFS (2.84,, 95%CI 1.87–4.31; Z = 4.92; P = 0.000). Additionally, the results of sensitivity analysis showed that the association was not changed after removing any study. Subgroup analysis revealed that both overexpressed HIF-1α and HIF-2α were associated with worse prognosis in CRC. On the basis of different HIF isoforms, further subgroup analysis was performed by study location, number of patients, antibody dilution, cut-off value. HIF-1α overexpression was significantly associated with poor OS in Asian countries (HR 2.3, 95% CI: 1.74–3.01, Z = 5.76, P = 0.000), while not in European or other countries. It indicated that HIF-1α overexpression seemed to be associated with disease progress and unfavorable prognosis in Asian CRC patients. Other factors did not alter the significant OS of overexpressed HIF-1α. In addition, significant correlations were observed between HIF-1α overexpression with clinicopathological features including Dukes' stages, UICC stages, depth of invasion, lymphnode status and metastasis. Our results concurred with previous study that HIF-1α expression had a significant inverse correlation in T1 and T2 CRC. On the other hand, overexpression of HIF-2α was significantly associated with grade of differentiation. Thus, HIF-1 and HIF-2 indicate distinct clinicopathologic features.

In this meta-analysis, we had dealt with highly significant heterogeneity among the 23 studies. Although we used random effects models to analyze the data, it did not identify the source of heterogeneity. Thus, we performed stratified analysis according to study location, number of patients, cut-off value. When the analysis on OS was performed without consideration of other factors, heterogeneity was detected (I^2^ 69.1% P = 0.000). While when the studies included were classified into three groups according to evaluation criterion (percentage, staining and percentage plus staining), the heterogeneity was not detected (I^2^ 0% P = 0.621, I^2^ 0% P = 0.624, I^2^ 0% P = 0.727). Therefore, the heterogeneity in this study could be explained by the evaluation standards. Meanwhile, there were some limitations in this meta-analysis. First, the study included in our meta-analysis was restricted only to articles published in English or Chinese, which probably provided additional bias. Second, HRs calculated from data or extracted from survival curves might be less reliable than direct analysis of variance. Third, the sample size in European studies was not big enough so that the difference of HIF-1expression on survival was not significant.

In summary, we showed that both overexpressed HIF-1α and HIF-2α were significantly associated with worse prognosis in CRC. Subgroup analysis indicated that HIF-1α overexpression was associated with progress disease and unfavorable prognosis in Asian CRC patients. Significant correlations were observed between HIF-1α overexpression with Dukes' stages, UICC stages, depth of invasion, lymphnode status and metastasis, but there was no significant association between overexpressed HIF-1α with grade of differentiation. While overexpressed HIF-2α was only associated with differentiation. Large, well-designed prospective studies are required to investigate the precise prognostic significance and clinicopathologic differences of HIFs expression.

## Supporting Information

Figure S1 **Egger's publication bias plot showed no publication bias for studies regarding overexpressed HIF-1α and overall survival (OS) in the meta-analysis: the relationship between the effect size of individual studies (HR, vertical axis) and the precision of the study estimate (standard error, horizontal axis).**
(TIF)

Figure S2 **Egger's publication bias plot showed no publication bias for studies regarding overexpressed HIF-1α and disease free survival (DFS) in the meta-analysis.**
(TIF)

Figure S3 **Egger's publication bias plot showed no publication bias for studies regarding overexpressed HIF-2α and overall survival (OS) in the meta-analysis.**
(TIF)

Checklist S1 **PRISMA Checklist.**
(DOC)
