# Supplementary figures and images for: Prognostic Value and Clinicopathological Differences of HIFs in Colorectal Cancer: Evidence from Meta-Analysis
Source: PLoS One. 2013 Dec 6;8(12):e80337. doi: 10.1371/journal.pone.0080337 (PMC3855620; doi:10.1371/journal.pone.0080337)

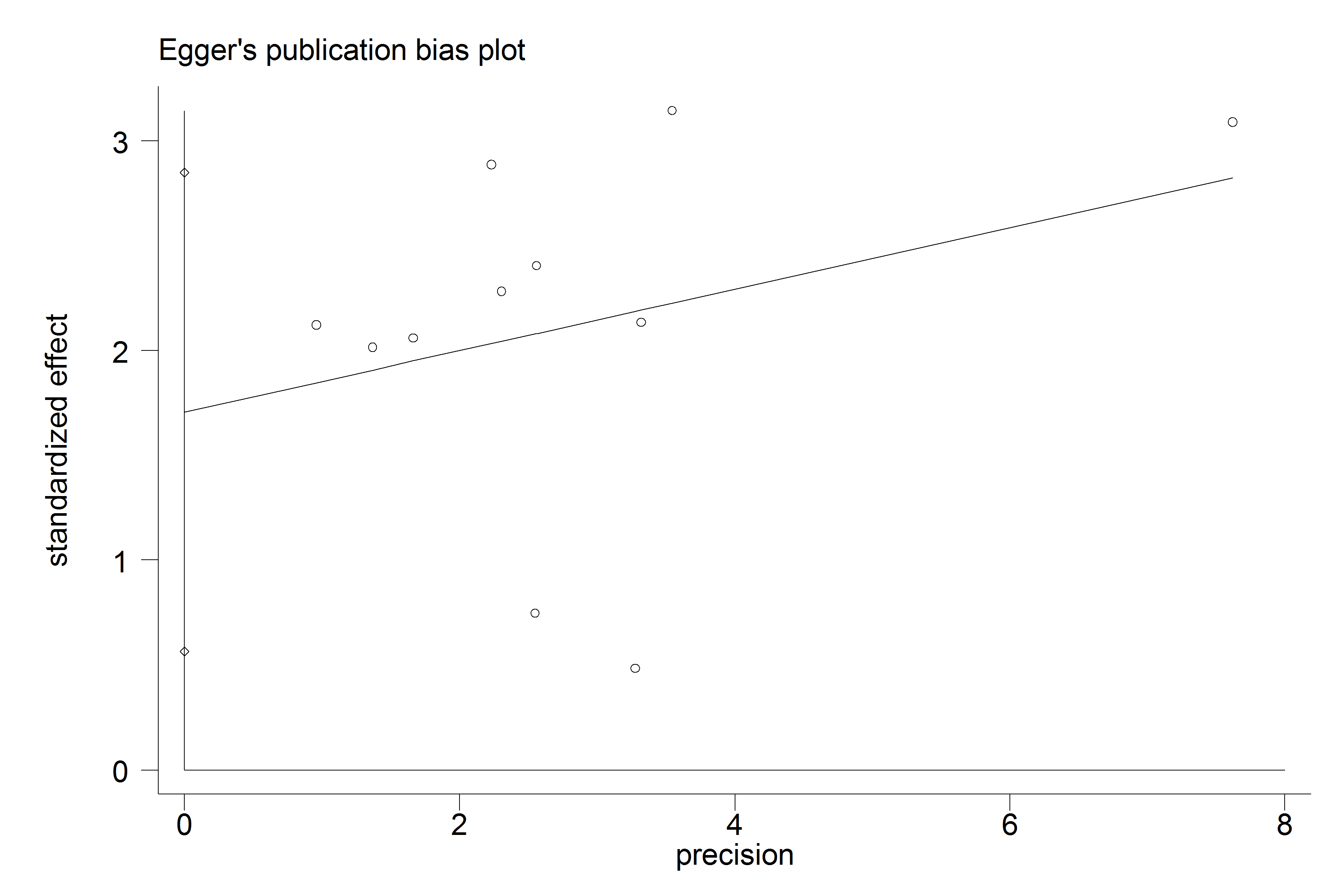

Supplement: Figure S1 — Egger's publication bias plot showed no publication bias for studies regarding overexpressed HIF-1α and overall survival (OS) in the meta-analysis: the relationship between the effect size of individual studies (HR, vertical axis) and the precision of the study estimate (standard error, horizontal axis). (TIF) [file pone.0080337.s001.tif]

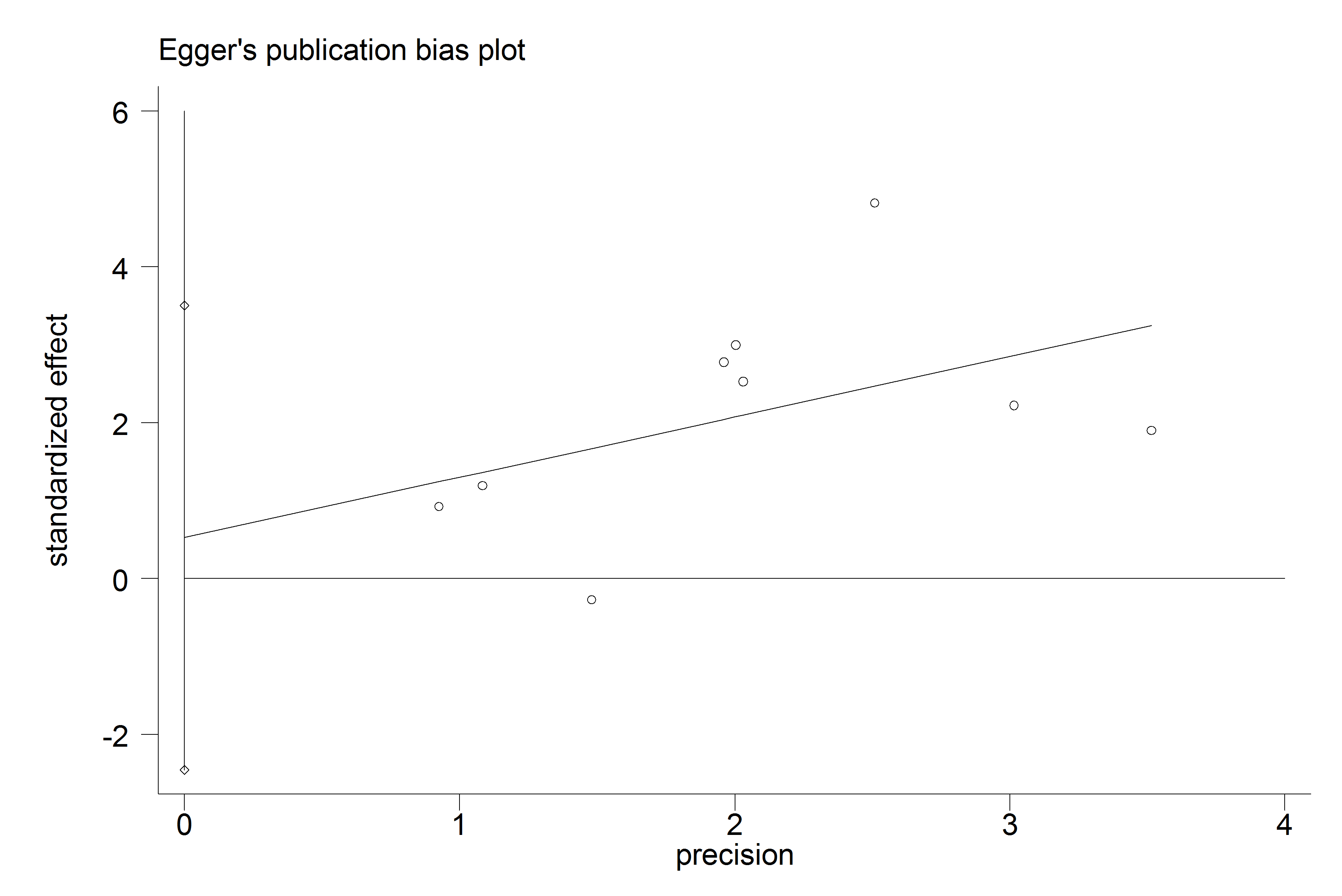

Supplement: Figure S2 — Egger's publication bias plot showed no publication bias for studies regarding overexpressed HIF-1α and disease free survival (DFS) in the meta-analysis. (TIF) [file pone.0080337.s002.tif]

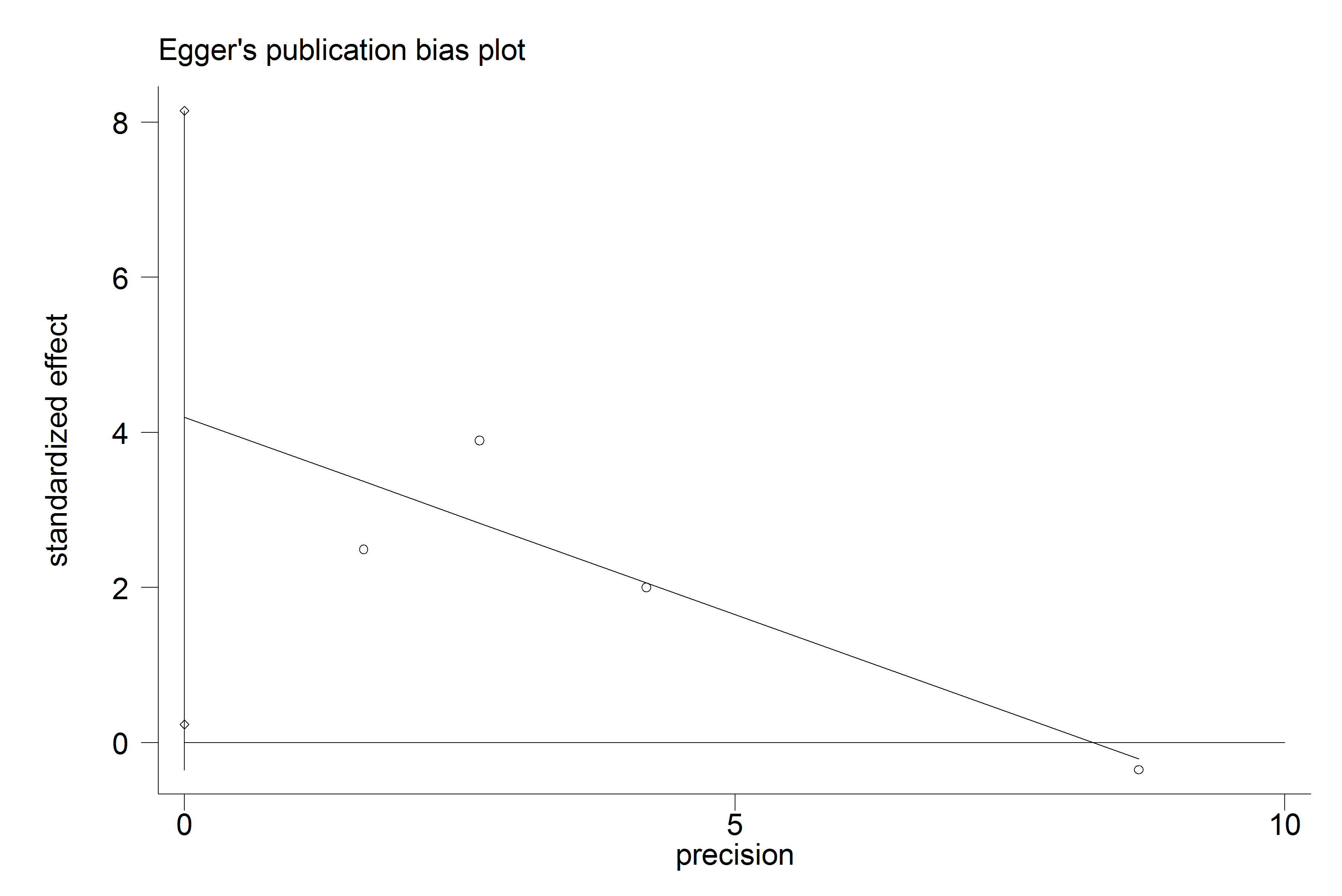

Supplement: Figure S3 — Egger's publication bias plot showed no publication bias for studies regarding overexpressed HIF-2α and overall survival (OS) in the meta-analysis. (TIF) [file pone.0080337.s003.tif]
